# Dual‐Pathway Defense: Ultramicro‐Pulverised Powder of *Polygonum chinense* L. and *Atractylodes rhizome* (PAUP) Rescues Broilers From *E. coli*‐Triggered Liver Injury Through Modulation of Oxidative Stress and Inflammation

**DOI:** 10.1002/fsn3.71424

**Published:** 2026-01-05

**Authors:** Jia‐Ci Cai, Yan‐Na Guo, Shao‐Shan Liang, Yan Liu, Fu‐Qiang Huang, Qi‐Peng Lv, Lan‐Yi Zhang, Yi Qin, Xiao‐Jing Chen, Yu‐Xin Liang, Yong‐Ming He, Lu‐Ping Tang

**Affiliations:** ^1^ School of Animal Science and Technology Foshan University Foshan China; ^2^ Xining Wildlife Park Xining Qinghai China

**Keywords:** broiler, *Escherichia coli*, liver injury, oxidative damage, ultramicro‐pulverized powder of *Polygonum chinense L*. and *Atractylodes rhizome* (PAUP)

## Abstract

This study characterizes the composition and blood components of ultramicro‐pulverized *Polygonum chinense* *L*. and *Atractylodes rhizome* powder (PAUP) and investigates its protective mechanisms against *Escherichia coli* ( *E. coli* )‐induced liver injury in broilers. PAUP constituents and serum metabolites were profiled by ultra‐high‐performance liquid chromatography‐mass spectrometry (UPLC‐MS). Fourteen‐day‐old broilers were randomly divided into 6 groups: control, *E. coli* model, gentamicin, and PAUP high/medium/low‐dose groups. Broilers were infected with 10^10^ CFU/mL *E. coli* O157:H7 via intraperitoneal injection. PAUP was administered 4 h post‐ *E. coli* infection for 7 days by gavage. Then, serum and liver samples from half of the chicks in each group were collected for further analysis. Recovery groups were analyzed after 7 additional days. UPLC‐MS identified 718 PAUP components, predominantly lipid, flavonoids, acids, and oxides. Serum metabolomics revealed 130 significantly elevated metabolites in PAUP groups, including atractylenolide I, linoleic acid, araliadiol, baicalin, gallocatechin, atractylodin, etc. PAUP normalized weight gain, reduced feed conversion ratio (FCR), and liver index in *E. coli* *‐*infected broilers. It significantly decreased bacterial load, suppressed serum AST and TBA levels, and relieved liver histopathological damage. Furthermore, PAUP restored redox balance (increased GSH activity, decreased MDA and ROS levels) via Nrf2‐HO‐1 pathway actvation (increased protein levels of Nrf2, Keap1, NQO1 and HO‐1). PAUP obviously attenuated inflammation (decreased IL‐6 and TNF‐α) through TLR4/NF‐κB inhibition. PAUP mitigates *E. coli*‐induced liver injury through Nrf2/HO‐1‐mediated antioxidant activation and TLR4/NF‐κB inflammatory suppression.

## Graphical abstract

Reduced Antibiotic residues and resistance concerns: Offers a potential natural alternative to antibiotics for combating *E. coli* infections in poultry. Provided safer poultry food products: By enhancing liver function, restoring weight gain, and improving feed efficiency, PAUP supports the production of healthier chickens, ultimately contributing to safer poultry food products and greater economic value for farmers.

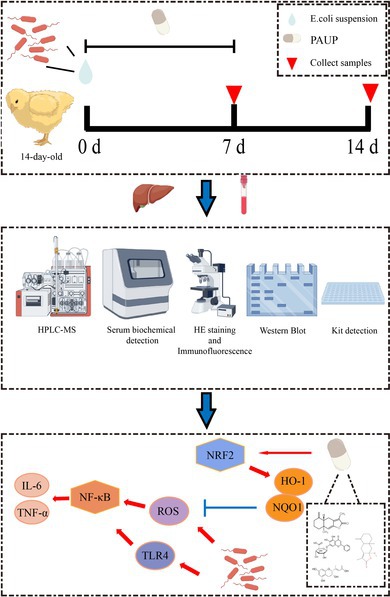

## 1 Introduction

*Escherichia coli* ( *E. coli* ) infection in broilers represents a significant threat to poultry health and food safety. As a major etiological agent of colibacillosis, septicemia, and hepatic or intestinal pathology, *E. coli* infection compromises flock performance, leading to reduced growth rates, increased mortality, and impaired productivity (Narasinakuppe Krishnegowda et al. 2022; Islam et al. 2023). These outcomes not only cause substantial economic losses but also heighten concerns related to the microbiological safety of poultry products entering the food chain. Pathogenic *E. coli* strains in poultry may contribute to carcass contamination, increasing the risk of foodborne illness in humans and posing a public health challenge. Furthermore, the persistence of infection and associated antimicrobial treatments can influence antimicrobial resistance dissemination, ultimately affecting human health and quality of life. Therefore, effective control and therapeutic strategies for *E. coli* in broilers are essential to safeguard animal welfare, ensure food safety, and protect public health.

The liver is a primary target organ affected by *E. coli* . Following entry into the bloodstream, *E. coli* can disseminate systemically, reaching organs such as the liver and resulting in hepatic inflammation, hepatomegaly, and abscess formation (Shen et al. 2023). However, research on *E. coli* ‐induced liver damage in broilers remains scare, and its underlying mechanisms are incompletely understood. Therefore, thoroughly elucidating the biological mechanism of liver damage in poultry during *E. coli* infection, along with identifying potential targets for prevention and treatment, provides critical guidance for developing drugs against avian colibacillosis. Currently, antibacterial drugs and vaccines are the primary effective treatments for *E. coli* infections. However, antibacterial drugs carry significant drawbacks, including variable side effects, resistance development, and drug residues, which pose serious public health risks (Barbarossa et al. 2022). Vaccines, meanwhile, lack broad efficacy due to the high genetic diversity and rapid mutation rate of *E. coli* strains (Pokharel et al. 2023). These limitations underscore the urgent need for alternative strategies to manage *E. coli* infections in poultry. Traditional Chinese medicine (TCM), with its lower residues and reduced side effects (Li et al. 2022), has gained attention in recent years as a potential alternative to conventional antibacterial agents.

Ultrafine powder technology is a cutting‐edge process that can make Chinese medicine into a powder with a particle size below 10 μm. It enhances the bioavailability of active ingredients, promotes the release of otherwise difficult‐to‐extract compounds, and improves the homogeneity and dissolution rate of powdered TCM products. Ultrafine powders can improve the stability and consistency of herbal formulations, ensuring more precise dosing and enhanced therapeutic outcomes (Zhi et al. 2019). Research on the application of TCM superfine powders in livestock and poultry is advancing. Research found that dietary rosemary ultrafine powder supplementation can enhance the health and productivity of aged hens (Li et al. 2024). Stems and leaves in ultrafined powder of Astragalus can boost the immune response to prevent Newcastle disease infections in poultry farms (Xi et al. 2014). Various studies will investigate their use as feed additives for purposes such as growth promotion, immune modulation, and enhancement of gut health, so as to explore their promising potential as natural alternatives or supplements (Guo et al. 2025).

*Atractylodes macrocephala* koidz (Baizhu) has been incorporated into traditional diets and medicinal cuisines for centuries, often used in porridges, soups, and tonics to strengthen the spleen and improve digestion. Studies show that its rhizomes and polysaccharides enhance metabolic status, strengthen immune responses, and reduce diarrhea and infection risks in animals (Lu et al. 2025; Rashidah et al. 2023). Its long history in both food therapy and herbal formulas highlights its role as a bridge between nourishment and medicinal health support. *Polygoni chinensis* L. is the dry ground part of Polygonaceae plant, and has the functions of soothing liver and improving eyes, clearing heat and dampness, cooling blood and detoxifying. It is widely used in the treatment of conditions such as dysentery, diarrhea and hepatitis (Hossen et al. 2015; Zeng et al. 2022). Modern pharmacological studies have demonstrated that *Polygoni chinensis* exhibits antibacterial and anti‐inflammatory effects, particularly in relation to liver health (OuYang et al. 2012). Our precious study has found that *Polygoni chinensis* L. upregulates IFN production to alleviate liver damage in mice caused by *Salmonella typhimurium* infection (Shen et al. 2022). *Polygoni chinensis* L. Capsules have a significant beneficial effect on liver fibrosis induced by carbon tetrachloride in rats (Dong et al. 2021). The two herbs can both be used to treat liver damage and colibacillosis. However, it is not clear whether the Ultramicro‐pulverized powder of *Polygoni chinensis* L. and *Atractylodes macrocephala koidz* can alleviate colibacillosis and its liver damage. Ultrafine powder of *Polygoni chinensis* L. and *Atractylodes macrocephala* Koidz. were mixed in a ratio of 1:4, which was named as PAUP. This study aims to investigate the effects of PAUP on liver damage in broilers with colibacillosis.

## 2 Materials and Methods

### 2.1 Bacteria

The *E. coli* O157:H7 strain was originally obtained from Guangdong Microbial Culture Collection Center (GDMCC NO:1.1869, Guangdong, China). The strain was inoculated onto lysogeny broth (LB) agar plate using an inoculation loop and cultured upside down in a constant temperature incubator at 37°C for 24 h. A single colony was then transferred to 15 mL of LB broth using an inoculation loop and incubated in a shaking incubator at 37°C and 220 rpm/min for 12 h to activate the strain. After activation, the culture was streaked on an agar plate and incubated upside down at 37°C for 12 h. Single colonies were selected and transferred to 15 mL of LB broth, incubated in a shaking incubator at 37°C and 220 rpm/min for 12 h. The culture was centrifuged at 5000 rpm/min for 10 min, and the bacteria were collected. The bacteria were washed with sterile PBS buffer and then re‐suspended it in sterile PBS buffer to obtain 10^10^ CFU/mL of *E. coli* O157:H7 bacterial suspension for further use.

### 2.2 Plant Material

Ultrafine powder technology was used to obtain the ultrafine powder of *Polygoni chinensis* L. and *Atractylodes macrocephala* Koidz. The two powders were then mixed in a ratio of 1:4 (PAUP), and distilled water was added to prepare a suspension. The final suspension concentrations were 150, 75, and 37.5 mg/mL, respectively.

### 2.3 Animals and Experimental Design

A total of 216 one‐day‐old Nanhai Mahuang chickens were raised to 14 days of age. Healthy broilers with comparable body weights were randomly divided into 6 groups: control, model, gentamicin (20 mg/kg), high‐dose PAUP (PAUP‐H, 1500 mg/kg), medium‐dose PAUP (PAUP‐M, 750 mg/kg), and low‐dose PAUP (PAUP‐L, 375 mg/kg). Each group consisted of 6 replicates with 6 birds per replicate. Broilers were infected with 1 mL of *E. coli* O157:H7 (10^10^ CFUs/chick) by intraperitoneal injection. Then, PAUP and gentamicin were subsequently administered by oral gavage for 7 continuous days, beginning 4 h post‐infection. Eighteen chicks from each group were anesthetized for blood collection via the jugular vein for serological assays and analysis of drug constituents. The remaining broilers were allowed to recover for an additional 7 days after treatment, after which liver samples were collected for organ index determination, H&E staining, and other analyses. Throughout the experiment, feed intake, body weight, clinical symptoms, and feces characteristics were recorded. The feed conversion ratio (FCR) and organ index of broilers were calculated using the formulas: FCR = Feed intake/weight gain. Organ index = (organ weight/broiler weight) × 100%. All broilers were raised with ordinary feeding; the composition and nutrient levels of the experimental diets were referred to previous article (Guo et al. 2024).

### 2.4 Serological Test

Serum samples were collected, and the concentrations of alkaline phosphatase (ALP, 140323004), albumin (ALB, 148322010), alanine aminotransferase (ALT, 140123007), aspartate aminotransferase (AST, 140223007), total bilirubin (T‐BilirV, 140,623,005), triglyceride (TG, 141723003), total cholesterol (TC, 2285099), total protein (TP, 140821003), and total bile acid (TBA, 143223006) were quantified using commercial assay kits following the manufacturer's instructions. All kits and the biochemical analyzer (BS‐240VET) were supplied by Shenzhen Mindray Bio‐Medical Electronics Co. Ltd. (Mindray, Shenzhen, China).

### 2.5 UPLC‐MS

50 mg of PAUP was transferred into a centrifuge tube, and 1 mL of extraction solution (water/acetonitrile/isopropyl alcohol, 1:1:1, v/v/v) was added to prepare the PAUP solution for UPLC‐MS analysis. Plasmam samples (0.5 mL) from broilers in the normal group and the PAUP‐treated group (1.5 g/kg) were collected after 7 days of drug administration. Extraction solvent (methanol/acetonitrile, 1: 1, v/v) was added to obtain plasmam PAUP extracts. All samples were sonicated at low temperature for 30 min and centrifuged at 12000 rpm for 10 min at 4°C. The resulting supernatants were stored at −20°C for 1 h to precipitate proteins, and then centrifuged again at 12000 rpm for 10 min at 4°C. The supernatant was vacuum‐dried and redissolved in 200 μL of 50% acetonitrile solution. After swirling, the samples were centrifuged at 14000 rpm for 15 min at 4°C, and the final supernatant was collected for analysis using a UPLC‐Q Exactive HFX system (Thermo, USA).

### 2.6 Bacterial Load

Hepatic bacterial load was quantified as follows: approximately 0.05 g of liver tissue was homogenized in 1 mL of sterile normal saline. Then, 100 μL of the homogenate was mixed with MacConkey agar plates and incubated at 37°C for 12 h to assess bacterial growth.

### 2.7 Tissue Histopathological Changes Examined by H&E Staining

The liver tissues were fixed in 4% paraformaldehyde for 24 h, and then subjected to hematoxylin and eosin (H&E) staining. Histopathological changes were examined using a light microscope (RVL‐100‐G, ECHO, America).

### 2.8 The Contents of GSH, MDA and SOD

A 10% liver tissue homogenate was prepared by adding 0.1 g of liver tissue to 0.9 mL of saline and homogenizing thoroughly. The homogenate was centrifuged at 2000 rpm for 10 min, and the supernatant was collected for biochemical analysis. Levels of malondialdehyde (MDA, 20220622), superoxide dismutase (SOD, 20230313), and glutathione (GSH, 20221028) were measured using commercial assay kits from Nanjing Jiancheng Bioengineering Institute (Nanjing, China).

### 2.9 Western Blot Analysis

Total protein was extracted from chicken liver using RIPA lysis buffer (1:100) supplemented with phenylmethylsulfonyl fluoroprotease inhibitor (PMSF). The proteins were separated by SDS–PAGE, transferred onto 0.22 μm PVDF membranes at low temperature, and blocked with 5% skim milk for 1 h at room temperature. Membranes were incubated for 3 h at room temperature with primary antibodies against TLR4 (#14358), NF‐κB (#8242), P‐NF‐κB (#3033), I‐κB (#4814), P‐I‐κB (#2859), IL‐6 (#16912), TNF‐α (#3707), Nrf2 (#12721), KEAP1 (#4678), NQO1 (#62262), HO‐1 (#86806), and β‐actin (#4970). After two 15 min washes with TBST, membranes were incubated with HRP‐conjugated sheep anti‐rabbit secondary antibody (SA00001‐2) for 1 h at room temperature, followed by two washes with TBST. Protein signals were visualized using ECLchemiluminescence, and band intensities were quantified using Image J software. All antibodies were obtained from Cell Signaling Technology (Massachusetts, American).

### 2.10 ROS Measurement

The frozen liver sections were gently air‐dried before adding ROS dye solution, followed by incubation at 37°C for 30 min in the dark. The sections were washed 3 times with PBS by shaking for 5 min each time. After slight drying, DAPI staining solution was applied and incubated at room temperature for 10 min. The sections were then dried again, mounted with anti‐fade medium, and examined using a fluorescence microscope (OLYMPUS IX53, Japan).

### 2.11 Statistical Analysis

Quantitative data are expressed as mean ± standard deviation. Statistical analysis and charting were performed using GraphPad Prism 8 (San Diego, CA, USA). Group differences were evaluated by one‐way nonparametric analysis of variance (ANOVA), and values of *p* < 0.05 were considered statistically significant.

## 3 Results

### 3.1 Analysis of Chemical Components of PAUP and Its Absorption Into Blood by UPLC‐MS

**FIGURE 1:**
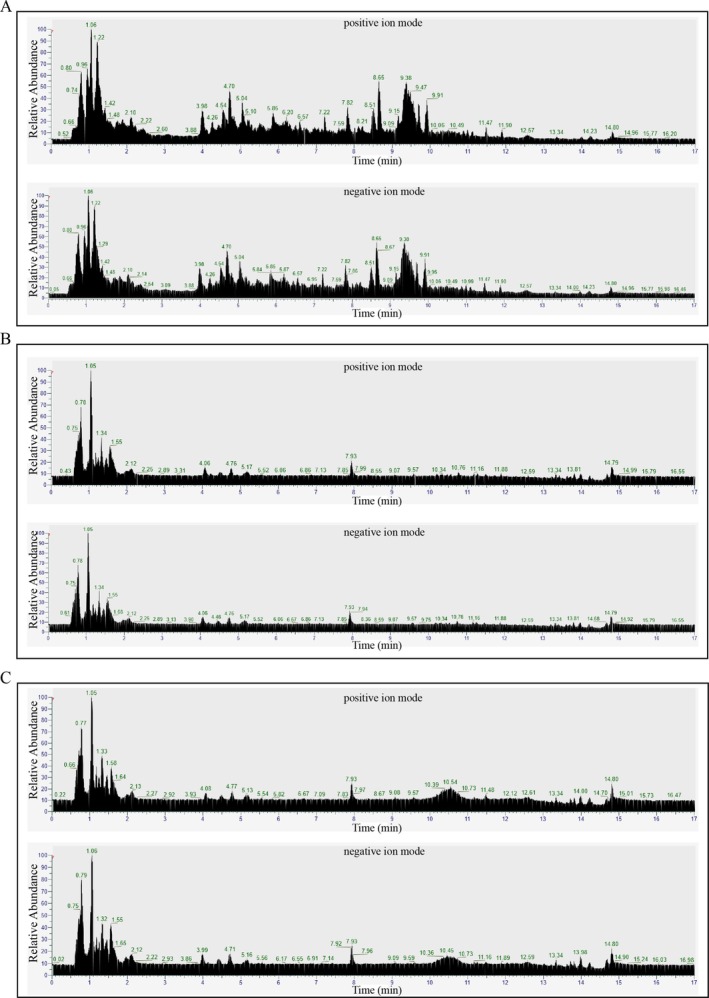
Chemical constituents of PAUP and blood analyzed by UPLC‐MS. (A) Chemical composition of PAUP. (B) Serum components in broilers of the control group. (C) Serum components in broilers of the PAUP‐H group.

As shown in Figure 1, a total of 718 components were detected in PAUP, including lipids, flavonoids, acids, oxides, and related metabolites. The top 30 compounds identified in the negative (NEG) and positive (POS) modes are listed in Tables 1 and 2, respectively. These major constituents included atractylenolide III, hesperidin, alpha‐lactose, D‐proline, citrate, and 3‐O‐caffeoylquinic acid, among others. Furthermore, comparative analysis of plasma samples from the control and PAUP‐H groups revealed that, compared to the control, 130 metabolites were significantly elevated in the PAUP‐H group (Table 3). These elevated metabolites included atractylenolide I, linoleic acid, araliadiol, baicalin, gallocatechin, and atractylodin.

**TABLE 1:** Chemical composition of PAUP (Positive ion mode).

Order	Metabolite	Formula	Retention time (min)	Proportion (%)
1	Hesperidin	C28H34O15	6.224	8.840
2	Citrate	C6H8O7	1.220	7.007
3	3‐O‐Caffeoylquinic acid	C16H18O9	4.285	6.385
4	2‐(9‐Decenyl) glutaconic acid	C15H24O4	9.692	5.281
5	Narirutin	C27H32O14	6.001	5.023
6	Ginnalin B	C13H16O9	2.127	4.442
7	3,5‐Dicaffeoylquinic acid	C25H24O12	6.165	4.328
8	Olivetol	C11H16O2	7.222	3.309
9	Phenylalanin	C9H11NO2	2.127	2.941
10	Chebulagic acid	C41H30O27	5.441	2.467
11	Didymin	C28H34O14	7.015	2.205
12	Cumanin	C15H22O4	8.866	1.814
13	Isomaltotriose	C18H32O16	0.819	1.667
14	Geraniin	C41H28O27	5.049	1.574
15	Chebulanin	C27H24O19	4.988	1.427
16	Epicatechin gallate	C22H18O10	5.883	1.268
17	(L)‐Dehydroascorbic acid	C6H6O6	1.293	1.261
18	Beta‐Glucogallin	C13H16O10	1.424	1.232
19	Neoschaftoside	C26H28O14	5.260	1.218
20	Oleuropeinic acid	C25H30O15	5.776	1.018
21	Orientin	C21H20O11	5.343	0.998
22	Di‐O‐methylbergenin	C16H20O9	5.224	0.854
23	1,6‐anhydro‐b‐D‐Glucose	C6H10O5	0.731	0.822
24	L‐Asparagine	C4H8N2O3	0.761	0.742
25	1,3‐Dicaffeoylquinic acid	C25H24O12	5.120	0.724
26	Vincosamide	C26H30N2O8	0.839	0.724
27	Cis‐1,2‐Dihydroxycyclohexane	C6H12O2	1.744	0.687
28	Azelaic acid	C9H16O4	6.552	0.686
29	Linoleic acid	C18H32O2	13.995	0.643
30	3‐hydroxy‐4‐E‐Hexenoic acid	C6H10O3	4.554	0.587

**TABLE 2:** Chemical composition of PAUP (Negtive ion mode).

Order	Metabolite	Formula	Retention time(min)	Proportion (%)
1	Atractylenolide III	C15H20O3	9.847	29.989
2	L‐Proline	C5H9NO2	0.803	11.324
3	Parthenolide	C15H20O3	9.300	4.451
4	Neochlorogenic acid	C16H18O9	4.653	4.264
5	Tangeretin	C20H20O7	9.869	4.211
6	Lathosterol	C27H46O	12.225	2.210
7	Byzantionoside B	C19H32O7	6.160	2.044
8	Vicenin 3	C26H28O14	5.189	1.622
9	Isoorientin	C21H20O11	5.271	1.549
10	1‐Linoleoyl‐sn‐glycero‐3‐phosphorylcholine	C26H50NO7P	10.767	1.405
11	Oxymatrine	C15H24N2O2	2.264	1.314
12	Curcumadione	C15H22O2	9.177	1.106
13	Aurantiamide acetate	C27H28N2O4	10.029	1.098
14	Naringenin	C15H12O5	5.919	0.940
15	Atractylenolide I	C15H18O2	11.659	0.911
16	Flavinantine	C19H21NO4	8.166	0.884
17	6‐Demethoxytangeretin	C19H18O6	8.776	0.876
18	Isosinensetin	C20H20O7	8.285	0.863
19	Mamanine	C15H22N2O2	2.124	0.783
20	1,3‐Di‐O‐caffeoylquinic aicd	C25H24O12	6.093	0.779
21	Sinensetin	C20H20O7	8.812	0.759
22	Isosakuranetin	C16H14O5	6.948	0.696
23	Eriodictiol‐7‐glucoside	C22H24O11	6.138	0.649
24	Kurarinone	C26H30O6	9.276	0.629
25	Salviolone	C18H20O2	10.587	0.600
26	Specnuezhenide	C31H42O17	5.835	0.591
27	Isovitexin	C21H20O10	5.650	0.578
28	Neoisoliquiritin	C21H22O9	5.919	0.545
29	Icariside B1	C19H30O8	6.354	0.522
30	Excavatin M	C19H20O7	9.363	0.505

**TABLE 3:** Blood components of PAUP.

Order	Metabolite	Formula	Order	Metabolite	Formula
1	Icosanedioic acid	C20H38O4	66	Obscuraminol F	C16H33NO
2	Linoleic acid	C18H32O2	67	Marumoside A	C14H19NO6
3	N‐Acetyl‐L‐ornithine	C7H14N2O3	68	(+)‐Junenol	C15H26O
4	Cyclo(Ile‐Leu)	C12H22N2O2	69	(2S,3R,4E)‐2‐Amino‐4‐heptadecene‐1,3‐diol	C17H35NO2
5	cis‐1,2‐Dihydroxycyclohexane	C6H12O2	70	n‐Docosanol	C22H46O
6	L‐Tyrosine	C9H11NO3	71	Stearic acid amide	C18H37NO
7	Isomaltotriose	C18H32O16	72	Obscuraminol B	C16H31NO
8	Ganoderal A	C30H44O2	73	Steviol‐19‐O‐Glucoside	C12H19N3O5
9	Methyl eugenol	C11H14O2	74	Triptobenzene H	C21H28O4
10	Abscisic Acid	C15H20O4	75	13,16,19‐Docosatrienoic acid	C22H38O2
11	Pelletierine	C8H15NO	76	D‐proline	C5H9NO2
12	L‐Asparagine	C4H8N2O3	77	Salirepin	C13H18O8
13	Bufalin	C24H34O4	78	Atractylodin	C13H10O
14	(R)‐a‐Terpinyl b‐D‐glucoside	C16H28O6	79	Betonicine	C7H13NO3
15	Furfuryl acetate	C7H8O3	80	9,10‐Methylenehexadecanoic acid	C17H32O2
16	Undecylenic acid	C11H20O2	81	Lycernuic ketone B	C31H48O6
17	1,11‐Undecanedicarboxylic acid	C13H24O4	82	Isoorientin	C21H20O11
18	Olivetol	C11H16O2	83	Tangeretin	C20H20O7
19	D‐Glucosamine	C6H13NO5	84	11‐Hydroxydrim‐7‐en‐6‐one	C15H24O2
20	4‐Hydroxyisoleucine	C6H13NO3	85	2,3,24‐Trihydroxy‐12‐ursen‐28‐oic acid	C30H48O5
21	Orientin‐2″‐O‐p‐trans‐coumarate	C30H26O13	86	Longicaulenone	C12H18O4
22	Chrysin‐7‐O‐glucuronide	C21H18O10	87	Calycosin	C16H12O5
23	Quinic acid	C7H12O6	88	Cholinesulfuric acid.	C5H13NO4S
24	2‐(9‐Decenyl)glutaconic acid	C15H24O4	89	6,9,10‐Trihydroxy‐7‐megastigmen‐3‐one	C13H22O4
25	Neoagarobiose	C12H20O10	90	6‐O‐Ethyltetradymodiol	C17H26O3
26	Caffeic acid	C9H8O4	91	Coniferaldehyde	C10H10O3
27	Shikimic acid	C7H10O5	92	Curcumol	C15H24O2
28	2‐O‐alpha‐D‐Glucopyranosyl‐L‐ascorbic acid	C12H18O11	93	Geranylacetone	C13H22O
29	4‐Hydroxy‐1,10‐secocadin‐5‐ene‐1,10‐dione	C15H24O3	94	Kaempferol	C15H10O6
30	Gamma‐Aminobutyric acid	C4H9NO2	95	6‐Shogaol	C17H24O3
31	Gentisic acid	C7H6O4	96	Cyclo(alanylleucyl)	C9H16N2O2
32	Isomangiferin	C19H18O11	97	Panowamycin B	C17H26O3
33	3‐hydroxy‐4‐E‐Hexenoic acid	C6H10O3	98	7,8‐Dihydroxyflavone	C15H10O4
34	Trans‐2,3‐Dihydro‐3‐ethoxyeuparin	C15H18O4	99	5‐Methoxyindoleacetate	C11H10NO3
35	Methyl Kakuol	C11H12O4	100	LINAMARIN	C10H17NO6
36	Vanillic acid	C8H8O4	101	Cucumegastigmane I	C13H20O4
37	6‐Hydroxy‐7‐methoxydihydroligustilide	C13H18O4	102	15‐Dihydroepioxylubimin	C15H26O3
38	N‐Acetyltryptamine	C12H14N2O	103	GeranylTiglate	C15H24O2
39	Aquilegiolide	C8H8O3	104	linolenic acid	C18H30O2
40	Umbelliferone	C9H6O3	105	Rhodiocyanoside A	C11H17NO6
41	Araliadiol	C15H20O2	106	Perillene	C10H14O
42	Baicalin	C21H18O11	107	Lysofungin	C27H49O12P
43	Sanggenol P	C30H36O6	108	(+)‐Perillaldehyde	C10H14O
44	Opuntiol	C7H8O4	109	Isoboonein	C9H14O3
45	3,4‐O‐Isopropylidene shikimic acid	C10H14O5	110	Homocitrulline	C7H15N3O3
46	Hymecromone	C10H8O3	111	Isoferulic acid	C10H10O4
47	3‐(hydroxymethyl)cyclopentanone	C6H10O2	112	6‐Demethoxytangeretin	C19H18O6
48	4‐Hydroxycinnamamide	C9H9NO2	113	1,3‐Di‐O‐caffeoylquinic aicd	C25H24O12
49	(−)‐Gallocatechin	C15H14O7	114	Trolline	C12H13NO3
50	Beta‐Glucogallin	C13H16O10	115	Betaine	C5H11NO2
51	Avicularin	C20H18O11	116	Senkyunolide I	C12H16O4
52	Vanillin	C8H8O3	117	Atractylenolide I	C15H18O2
53	Ethyl gallate	C9H10O5	118	Excavatin M	C19H20O7
54	Griffonilide	C8H8O4	119	Prunasin acid	C14H18O8
55	Myriocin	C21H39NO6	120	4‐Methylumbelliferone	C10H8O3
56	Gallic acid	C7H6O5	121	Protocatechualdehyde	C7H6O3
57	Ethyl 2,4,6‐trihydroxybenzoate	C9H10O5	122	Shizukanolide C	C15H18O3
58	3‐O‐Caffeoylquinic acid	C16H18O9	123	Songoramine	C22H29NO3
59	Shizukanolide H	C17H20O5	124	Benzyl glucopyranoside	C13H18O6
60	Esculetin	C9H6O4	125	Neridienone B	C21H28O4
61	Loganetin	C11H16O5	126	Norpterosin B	C13H16O2
62	15‐Nor‐14‐oxolabda‐8(17),12‐dien‐18‐oic acid	C19H28O3	127	Alpha‐Cembrenediol	C20H34O2
63	Brevifolincarboxylic acid	C13H8O8	128	Cis‐3,4‐Dihydroxy‐beta‐ionone	C13H20O3
64	Diosmetin	C16H12O6	129	2,2,5,7,8‐Pentamethyl‐6‐Chromanol	C14H20O2
65	DL‐Lysine	C6H14N2O2	130	3‐Butylidenephthalide	C12H12O2

### 3.2 PAUP Increased Growth Performance in Broilers Infected With *E. coli*

**FIGURE 2:**
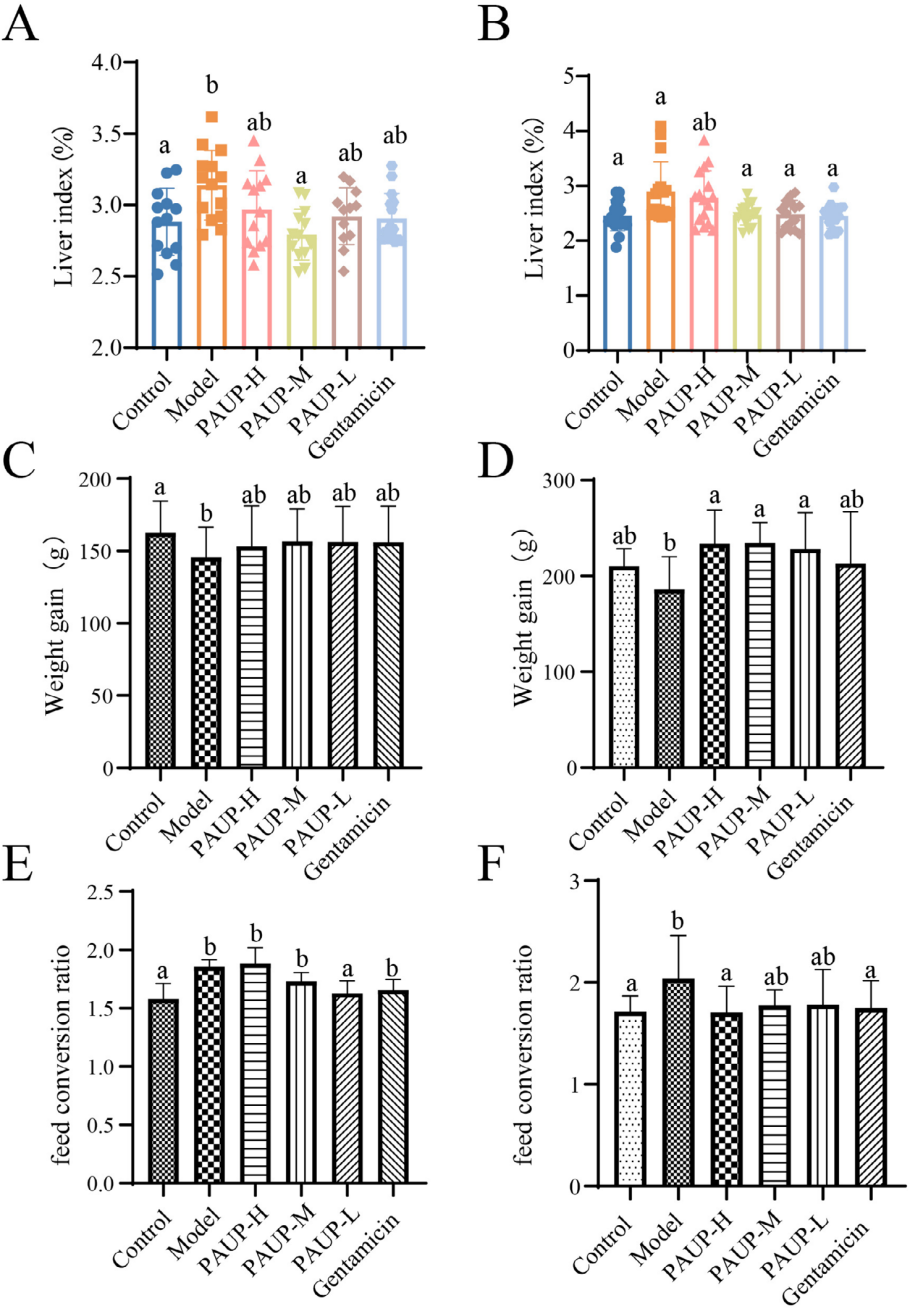
Effects of PAUP on growth performance in broilers infected with *E. coli* . (A) Liver index of broilers during the drug treatment period. (B) Liver index of broilers to recover for an additional 7 days after drug treatment. (C) weight gain, and (E) feed conversion ratio (FCR) of broilers during the drug treatment period. (D) weight gain and (F) FCR of broilers to recover for an additional 7 days after drug treatment. Bars with the same letter indicate no significant difference (*p* > 0.05), while bars with different letters indicate a significant difference (*p* < 0.05).

As shown in Figure 2C–F *E. coli* infection significantly reduced body weight gain and increased the feed conversion ratio (FCR), indicating impaired growth performance. FCR was lower in the PAUP‐L group compared with the model group (*p* < 0.05), whereas gentamicin did not produce a notable improvement. After a 7‐day recovery period, broilers in the PAUP‐treated groups exhibited significantly increased weight gain and decreased FCR compared to the model group. These results demonstrate that PAUP improved the growth performance in *E. coli* ‐infected broilers, with its growth‐promoting effects persisting for at least 1 week following treatment withdrawal.

The liver index was significantly elevated in the model group compared with the control group (*p* < 0.05, Figure 2A), but this increase was attenuated by PAUP‐M treatment (*p* < 0.05). After the 7‐day recovery period, liver index in all drug‐treated groups remained unchanged relative to measurements taken at the end of the treatment period.

### 3.3 PAUP Alleviated Alterations in Serological Enzymatic Activity in Broilers Infected With *E. coli*

Compared with the control group, the model group exhibited significantly increased serum aspartate aminotransferase (AST) and total bile acid (TBA) levels (*p* < 0.05), accompanied by a significant reduction in triglyceride (TG) levels (*p* < 0.05, Table 4). Administration of PAUP attenuated these alterations across all measured indices relative to the model group.

**TABLE 4:** Serum biochemical of broilers.

	Control	Model	PAUP‐H	PAUP‐M	PAUP‐L	Gentamicin	Recovery control	Recovery model	Recovery PAUP‐H	Recovery PAUP‐M	Recovery PAUP‐L	Recovery gentaminic
ALB	13.59 ± 1.38^abc^	13.99 ± 0.94^bc^	13.29 ± 1.09^abc^	13.01 ± 1.09^ab^	12.84 ± 1.40^a^	14.17 ± 1.69^c^	13.16 ± 1.18^a^	14.92 ± 1.71^b^	13.31 ± 1.57^a^	13.37 ± 1.48^a^	13.38 ± 1.37^a^	13.04 ± 1.77^a^
ALP	2352.75 ± 775.57^a^	3351.52 ± 1590.18^ab^	3439.22 ± 2023.33^ab^	3455.68 ± 2001.53^ab^	3983.40 ± 1752.95^b^	4286.78 ± 1913.78^b^	3127.04 ± 2021.36^a^	2419.95 ± 1437.65^a^	3420.24 ± 1718.76^ab^	4440.20 ± 1413.56^b^	3125.94 ± 1709.21^a^	3062.57 ± 2203.74^a^
ALT	2.17 ± 0.78^ac^	2.91 ± 0.61^bc^	2.40 ± 0.73^ab^	2.67 ± 0.56^abc^	2.59 ± 0.70^abc^	3.16 ± 0.77^c^	2.68 ± 0.76^a^	2.90 ± 0.78^a^	2.84 ± 0.74^a^	2.56 ± 0.96^a^	3.22 ± 0.70^a^	2.94 ± 0.59^a^
AST	222.12 ± 23.32^a^	290.10 ± 97.14^b^	213.95 ± 18.77^a^	225.73 ± 29.66^a^	222.18 ± 26.83^a^	215.16 ± 22.90^a^	202.49 ± 20.84^acd^	206.32 ± 20.56^acd^	187.18 ± 21.00^b^	208.05 ± 14.76^c^	193.51 ± 17.13^d^	185.45 ± 17.94^b^
TBA	3.37 ± 1.17^a^	5.24 ± 2.40^b^	3.25 ± 0.79^a^	3.15 ± 0.71^a^	3.03 ± 0.67^a^	3.73 ± 1.83^a^	4.97 ± 1.87^acd^	3.51 ± 1.28^bc^	5.00 ± 2.60^acd^	4.19 ± 1.34^c^	5.73 ± 2.75^d^	4.10 ± 1.47^ab^
T‐BIL	18.59 ± 2.94^a^	21.06 ± 2.89^a^	18.87 ± 3.34^a^	19.38 ± 3.64^a^	19.96 ± 2.83^a^	23.71 ± 3.32^b^	5.52 ± 1.58^ab^	3.29 ± 1.15^b^	4.82 ± 2.90^ab^	5.52 ± 2.23^a^	5.91 ± 1.88^a^	5.35 ± 1.98^ab^
TC	3.56 ± 0.39^a^	3.84 ± 0.41^a^	3.60 ± 0.25^a^	3.51 ± 0.34^a^	3.59 ± 0.35^a^	4.01 ± 0.37^b^	3.49 ± 0.42^a^	3.31 ± 0.34^a^	3.43 ± 0.44^a^	3.53 ± 0.37^a^	3.47 ± 0.51^a^	3.53 ± 0.35^a^
TG	0.31 ± 0.06^a^	0.30 ± 0.03^a^	0.34 ± 0.05^ab^	0.32 ± 0.06^a^	0.34 ± 0.04^ab^	0.31 ± 0.05^b^	0.31 ± 0.07^a^	0.30 ± 0.09^a^	0.31 ± 0.08^a^	0.29 ± 0.06^a^	0.31 ± 0.07^a^	0.32 ± 0.07^a^

*Note:* Bars with the same letter indicate no significant difference (*p* > 0.05), while bars with different letters indicate a significant difference (*p* < 0.05).

### 3.4 PAUP Reduced Liver Bacterial Load and Histopathological Damage in Broilers Infected With *E. coli*

**FIGURE 3:**
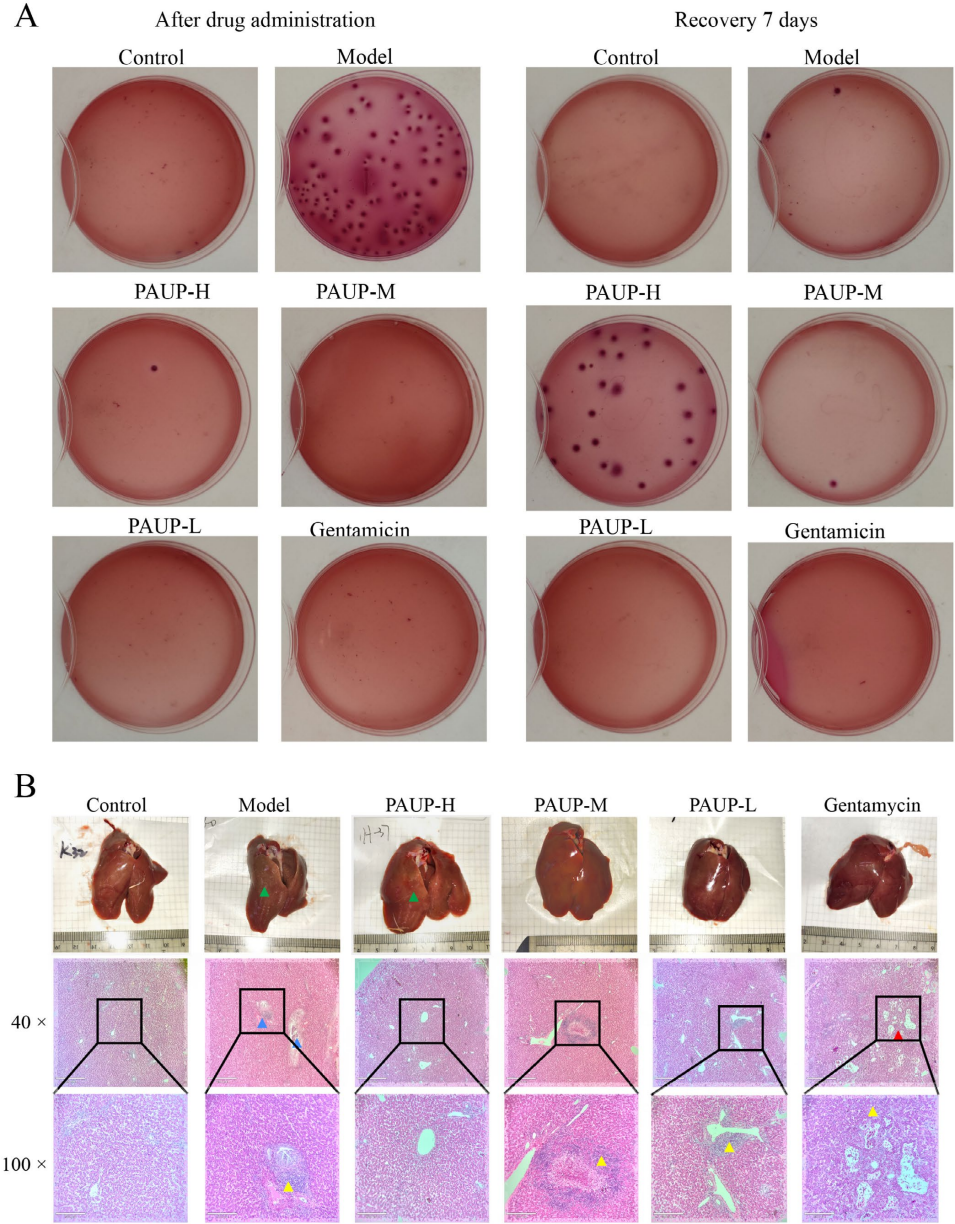
Effects of PAUP on liver bacterial load and histopathological changes in broilers infected with *E. coli* . (A) Liver bacterial load. (B) Liver histopathological changes examined by H&E staining. Blue triangles indicate liver abscesses, yellow triangles indicate inflammatory cell infiltration, and red triangles indicate hepatic vacuolation.

As shown in Figure 3A *E. coli* infection resulted in a marked increase in hepatic bacterial load. This elevation was markedly reduced in all PAUP‐treated groups as well as in the gentamicin group. By day 7 of the recovery period, bacterial counts were undetectable in both the PAUP‐L and gentamicin groups.

*E. coli* infection induced visible white necrotic spots on the liver surface and evident hepatic abscesses under microscopic examination (Figure 3B). Histopathological assessment revealed fibrous tissue proliferation, congestion, inflammatory cell infiltration, and central necrosis with hepatocellular degeneration. In the PAUP‐H group, only occasional white necrotic spots and mild microvascular congestion were observed. The PAUP‐M group showed no gross abnormalities macroscopically, although mild hepatic abscesses were detected microscopically and were notably less severe than those in the model group. No macroscopic lesions were detected in the PAUP‐L group. However, histological examination demonstrated loosely arranged hepatocytes and mild perivascular inflammatory cell infiltration compared with the control group. In the gentamicin‐treated group, liver abscesses were absent, but hepatocytes displayed ballooning degeneration.

### 3.5 PAUP Alleviated Liver Oxidative Damage in Broilers Infected With *E. coli*

**FIGURE 4:**
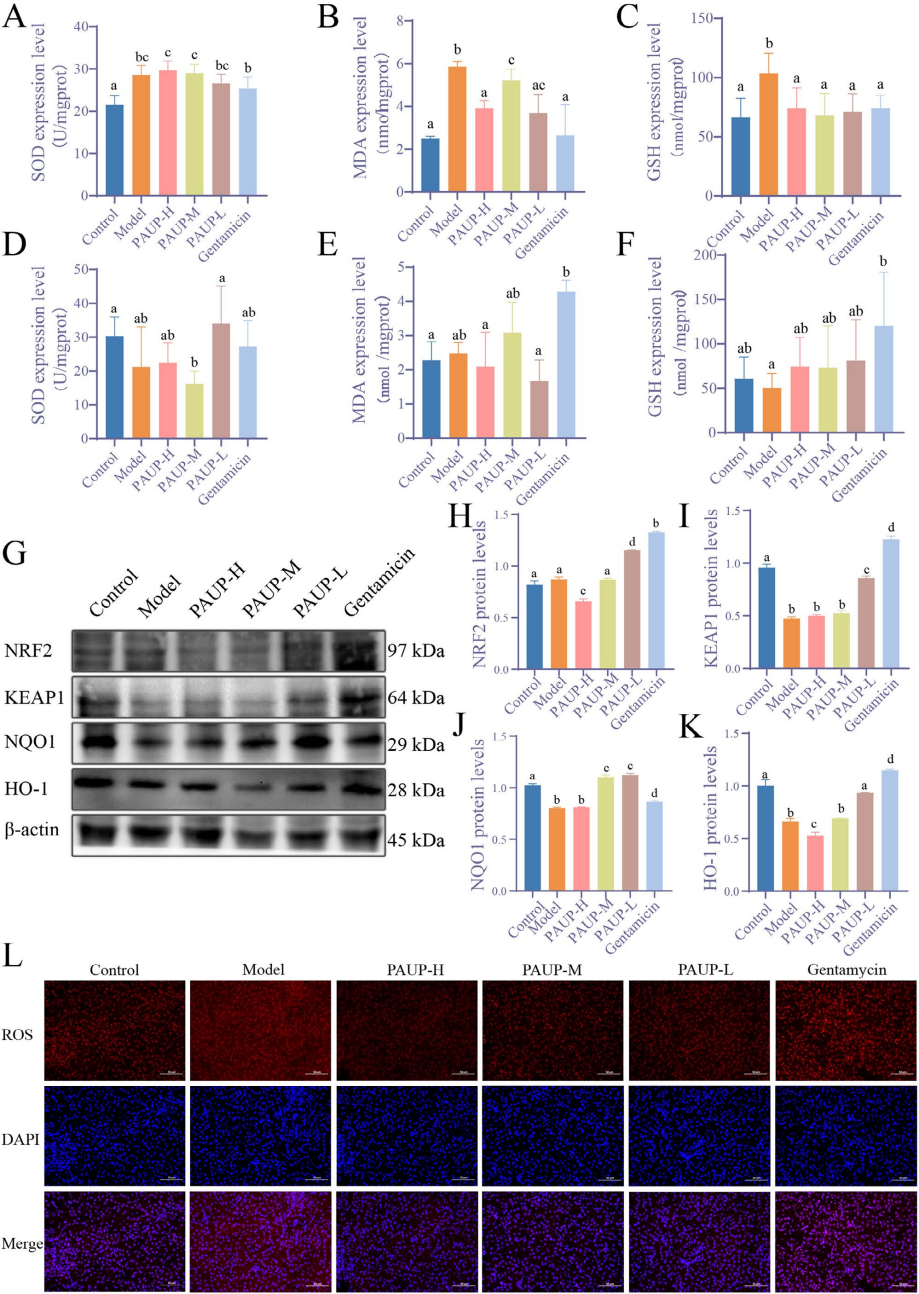
Effects of PAUP on oxidative damage in liver of broilers infected with *E. coli* . (A–C) Hepatic levels of SOD, MDA, and GSH following drug administration. (D, E) Hepatic SOD, MDA, and GSH levels after 7 days of recovery. (G–K) Protein expression of factors related to the Nrf2‐Keap1 signaling pathway. (L) Hepatic ROS expression detected by immunofluorescence.

Compared with the control group, *E. coli* infection significantly elevated hepatic levels of SOD, MDA, and GSH (*p* < 0.05, Figure 4 A‐C). PAUP treatment markedly reduced MDA and GSH concentrations (*p* < 0.05). After a 7‐day recovery period, hepatic SOD, GSH, and MDA levels in all treated groups returned to values comparable to those of the control group (*p* > 0.05, Figure 4D–F).

Hepatic ROS levels were significantly elevated in the model group relative to the control (Figure 4L). PAUP treatment significantly reduced ROS levels, whereas gentamicin produced no significant effect. Analysis of the NRF2‐KEAP1 signaling pathway revealed that protein expression levels of KEAP1, NAD(P)H quinone dehydrogenase 1 (NQO1), and heme oxygenase‐1 (HO‐1) were significantly decreased in the model group relative to the control group (Figure 4G–K). PAUP treatment significantly upregulated NRF2, KEAP1, NQO1, and HO‐1 protein expression, with the robust activation observed in the PAUP‐L group (*p* < 0.05). These results indicate that PAUP mitigates hepatic oxidative damage by enhancing the NRF2‐KEAP1 antioxidant signaling pathway.

### 3.6 PAUP Alleviated Liver Inflammatory Inhury in Broilers Infected With *E. coli*

**FIGURE 5:**
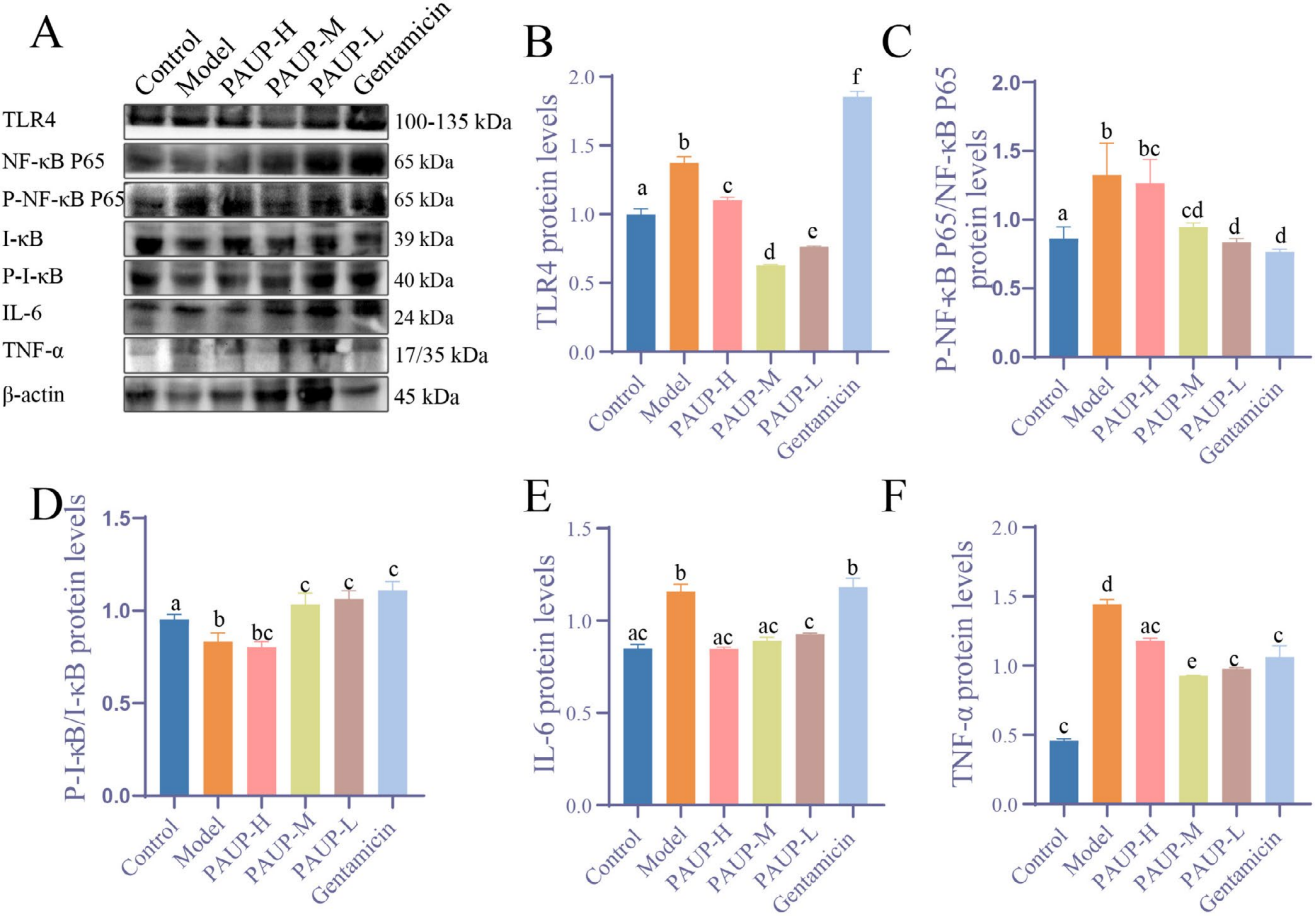
Effects of PAUP on the TLR4/NF‐κB signaling pathway in the liver of broilers infected with *E. coli* . (A–F) Protein expression levels of TLR4, NF‐κB, p‐NF‐κB, IκB, p‐IκB, IL‐6, and TNF‐α detected by Western Blot.

Compared with the control group, the model group exhibited significantly elevated protein expression levels of TLR4, IL‐6, and TNF‐α, along with increased phosphorylation of NF‐κB p65 (reflected by the P‐NF‐κB p65/NF‐κB p65 ratio) and decreased phosphorylation of I‐κB (P‐I‐κB/I‐κB ratio) (*p* < 0.05, Figure 5). Treatment with PAUP‐M and PAUP‐L significantly attenuated these inflammatory alterations (*p* < 0.05), demonstrating stronger anti‐inflammatory effects than gentamicin. These results indicate that PAUP mitigates hepatic inflammatory damage in *E. coli* –infected broilers, through suppression of the TLR4/NF‐κB signaling pathway.

## 4 Discussion

In this study, UPLC‐MS was used to analyze the chemical composition of PAUP and to identify its serum metabolites in broilers. A total of 718 constituent compounds were detected, predominantly lipid molecules, flavonoids, phenolic acids, and their oxidized derivatives. Following oral administration, 130 compounds exhibited elevated serum levels compared to controls. Key circulating metabolites included neoagarobiose, araliadiol, baicalin, sanggenol P, (−)‐Gallocatechin, 7,8‐Dihydroxyflavone, 6‐demethoxytangeretin, excavatin M, atractylenolide I, and benzyl derivatives. Previous studies have shown that several of these PAUP constituents possess antioxidant, anti‐inflammatory, and antimicrobial bioactivities. For example, baicalin demonstrates inhibitory effects against *E. coli* isolated from bovine mastitic milk and reduces antimicrobial resistance (Zhao et al. 2018). It also suppresses co‐infection by *Mycoplasma gallisepticum* and *E. coli* in chickens by inhibiting the NF‐κB signaling pathway (Wu et al. 2020). (−)‐Gallocatechin demonstrates antioxidant activity through inhibition of the phosphorylation of ERK and JNK (Park et al. 2021). Our previous work demonstrated that *E. coli* O157:H7 infection induces hepatic colonization, oxidative damage, and inflammatory responses in broilers with colibacillosis (Guo et al. 2024). In the present study, histopathological assessment similarly revealed multifocal necrotic spots, hepatic abscess formation, vascular congestion, and inflammatory cell infiltration in infected livers. PAUP administration significantly attenuated these pathological alterations. Furthermore, PAUP‐treated broilers exhibited improved growth performance, reduced liver bacterial burden, decreased serum AST and TBA concentrations, and attenuated liver tissue injury. These findings demonstrate the therapeutic potential of PAUP for mitigating *E. coli* ‐induced hepatic injury in broilers and support its development as a promising alternative or adjunct to conventional antimicrobial therapies.

Upon *E. coli* invasion, disruption of redox homeostasis within the host organism triggers pronounced oxidative stress (Yu et al. 2024). Mitochondria are the primary source of reactive oxygen species (ROS), and the liver contains a high density of mitochondria; it is a principal target organ vulnerable to ROS‐mediated oxidative damage (Zhang et al. 2022). In this study, *E. coli* infection induced evident hepatic injury accompanied by notably elevated intracellular ROS levels in hepatocytes. GSH, a key endogenous antioxidant, is synthesized in response to increased ROS to mitigate oxidative damage (Niu et al. 2021). The significantly increased GSH levels observed in *E. coli* ‐infected broilers suggested a compensatory antioxidant response to oxidative challenge. However, persistently high GSH concentrations may also indicate ongoing oxidative burden and compromised antioxidant capacity. PAUP treatment significantly attenuated hepatic ROS accumulation and restored GSH concentrations to baseline, demonstrating its capacity to alleviate oxidative stress and reestablish redox homeostasis.

Nrf2 serves as a master regulator of cellular defense against oxidative stress by orchestrating the transcription of numerous genes involved in maintaining redox homeostasis (He et al. 2020). One of its key downstream effectors is HO‐1, a stress‐inducible enzyme with potent antioxidant and cytoprotective properties (Zhang et al. 2021). Previous studies have demonstrated that 7,8‐dihydroxyflavone exerts antioxidant effects via induction of HO‐1 expression and suppression of caspase‐3/PARP activation (Chen et al. 2024). Atractylenolide‐I attenuates oxidative stress and Parkinson's disease–related pathology by activating the SIRT1/PGC‐1α/Nrf2 signaling axis (Gao et al. 2024). In the present study, PAUP significantly enhanced the Nrf2/HO‐1 signaling pathway, as demonstrated by the upregulated expression of Nrf2 and its downstream antioxidant proteins, including Keap1, NQO1, and HO‐1. This activation plays a critical role in strengthening the cellular defense system against oxidative stress. Moreover, PAUP treatment significantly reduced hepatic ROS levels, further indicating its protective effect against oxidative damage. These findings demonstrate that PAUP not only mitigates the acute oxidative burden induced by *E. coli* infection but also activates key endogenous defense mechanisms, thereby bolstering cellular resilience to oxidative injury.

Nuclear factor kappa‐B cells (NF‐κB) is essential for mediating immune responses to bacterial infections (Khan et al. 2024). Its activation can proceed through the canonical pathway, characterized by I‐κB phosphorylation and degradation, followed by nuclear translocation of the p65 subunits or through noncanonical mechanisms. Lipopolysaccharide (LPS) engages TLR4 and activates NF‐κB via MyD88, while ROS can also regulate NF‐κB activation (Sun 2017). Atractylenolide I has been shown to alleviate APAP‐induced hepatic oxidative damage through modulation of the TLR4/MAPKs/NF‐κB axis (Du et al. 2022). In the present study, *E. coli* infection activated NF‐κB predominantly through upregulation of TLR4 and accumulation of ROS, rather than through increased I‐κB phosphorylation, leading to elevated production of IL‐6 and TNF‐α. PAUP significantly attenuated TLR4 expression and ROS levels, thereby suppressing NF‐κB signaling and alleviating liver inflammation in broilers.

## 5 Conclusions

These results demonstrate that PAUP concurrently modulates both the TLR4/NF‐κB signaling and the Nrf2 antioxidant pathway, thereby establishing a comprehensive protective mechanism that effectively counteracts *E. coli* ‐induced liver damage in broilers. These findings highlight PAUP's potential to improve poultry health and food safety, reducing bacterial contamination and oxidative damage. This study provides a scientific basis for translating such natural interventions to support human health through safer, more nutritious animal‐derived food products.

## Author Contributions

Conceptualization and investigation and funding: Lu‐Ping Tang; Conceptualization: Yong‐Ming He; Investigation and methodology and writing – original draft: Jia‐Ci Cai, Yan‐Na Guo and Shao‐Shan Liang: Formal analysis and supervision and software, Yan Liu, Fu‐Qiang Huang, and Qi‐Peng Lv: Data curation and resources: Lan‐Yi Zhang, Yi Qin, Xiao‐Jing Chen and Yu‐Xin Liang. All authors have read and agreed to the published version of the manuscript.

## Funding

National Natural Science Foundation of China [Grant No. 32402930]. Guangdong Provincial Department of Science and Technology [Grant No. 2024A1515030170]. Department of Education of Guangdong Province [Grant No. 2024KTSCX208]. Science and Technology Projects of Xizang Autonomous Region, China [Grant No. XZ202501ZY0122].

## Ethics Statement

All animal work is carried out in accordance with the Guidelines for the Care and Use of Laboratory Animals formulated by the Ministry of Science and Technology, PRC (approval No.: 2006–398) and approved by the Laboratory Animal Management Committee of Foshan University. All efforts were made to minimize animal suffering.

## Conflicts of Interest

The authors declare no conflicts of interest.

## Data Availability

All data supporting the findings of this study are included in the article and its Supporting Information. No additional datasets were generated or analyzed.
